# Supplementary figures and images for: An Integrated Analysis of microRNAs and the Transcriptome Reveals the Molecular Mechanisms Underlying the Regulation of Leaf Development in Xinyang Maojian Green Tea (Camellia sinensis)
Source: Plants (Basel). 2023 Oct 24;12(21):3665. doi: 10.3390/plants12213665 (PMC10649745; doi:10.3390/plants12213665)

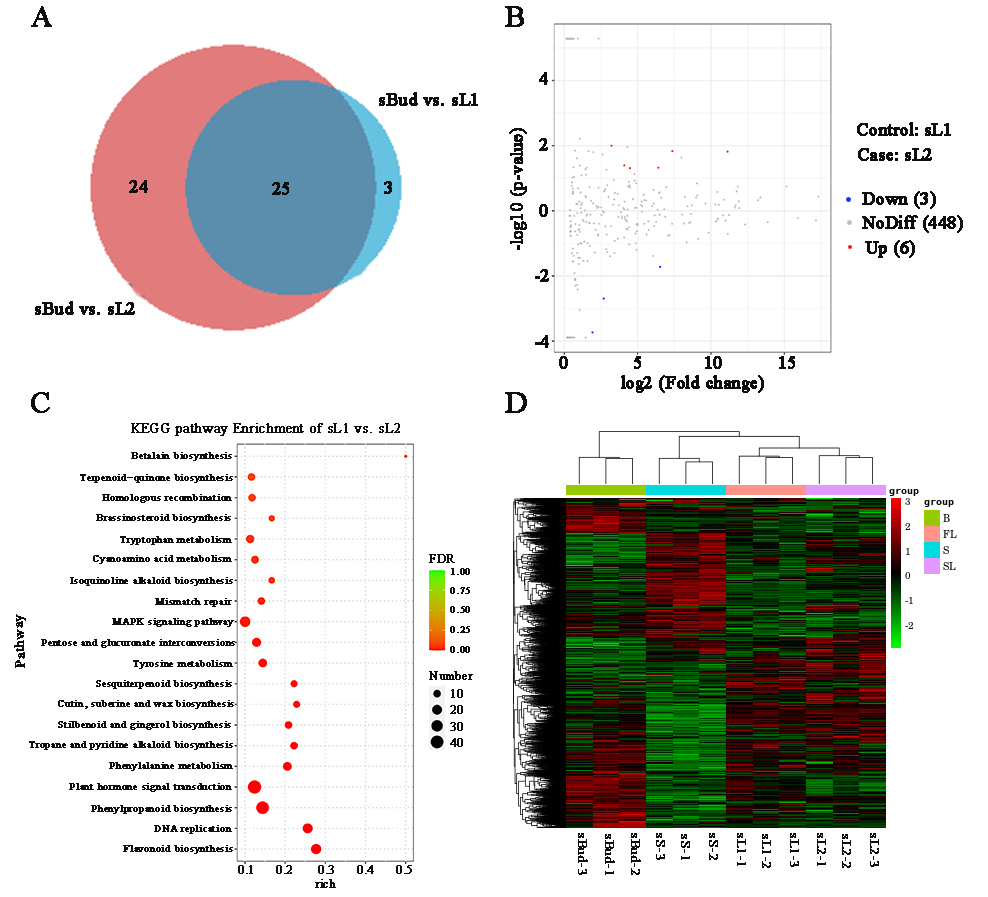

Supplement: Supplementary file 1 [file plants-12-03665-s001.zip › Figure S1.jpg]

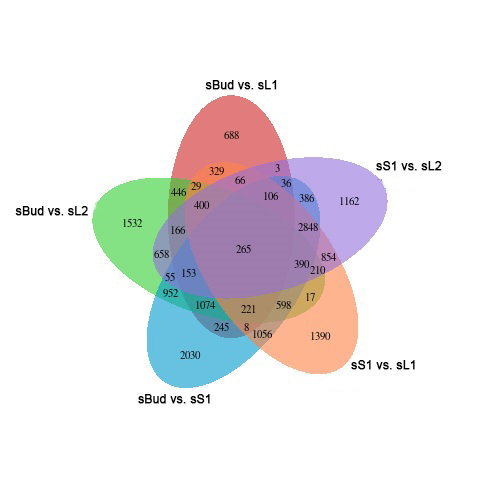

Supplement: Supplementary file 1 [file plants-12-03665-s001.zip › Figure S2.JPEG]

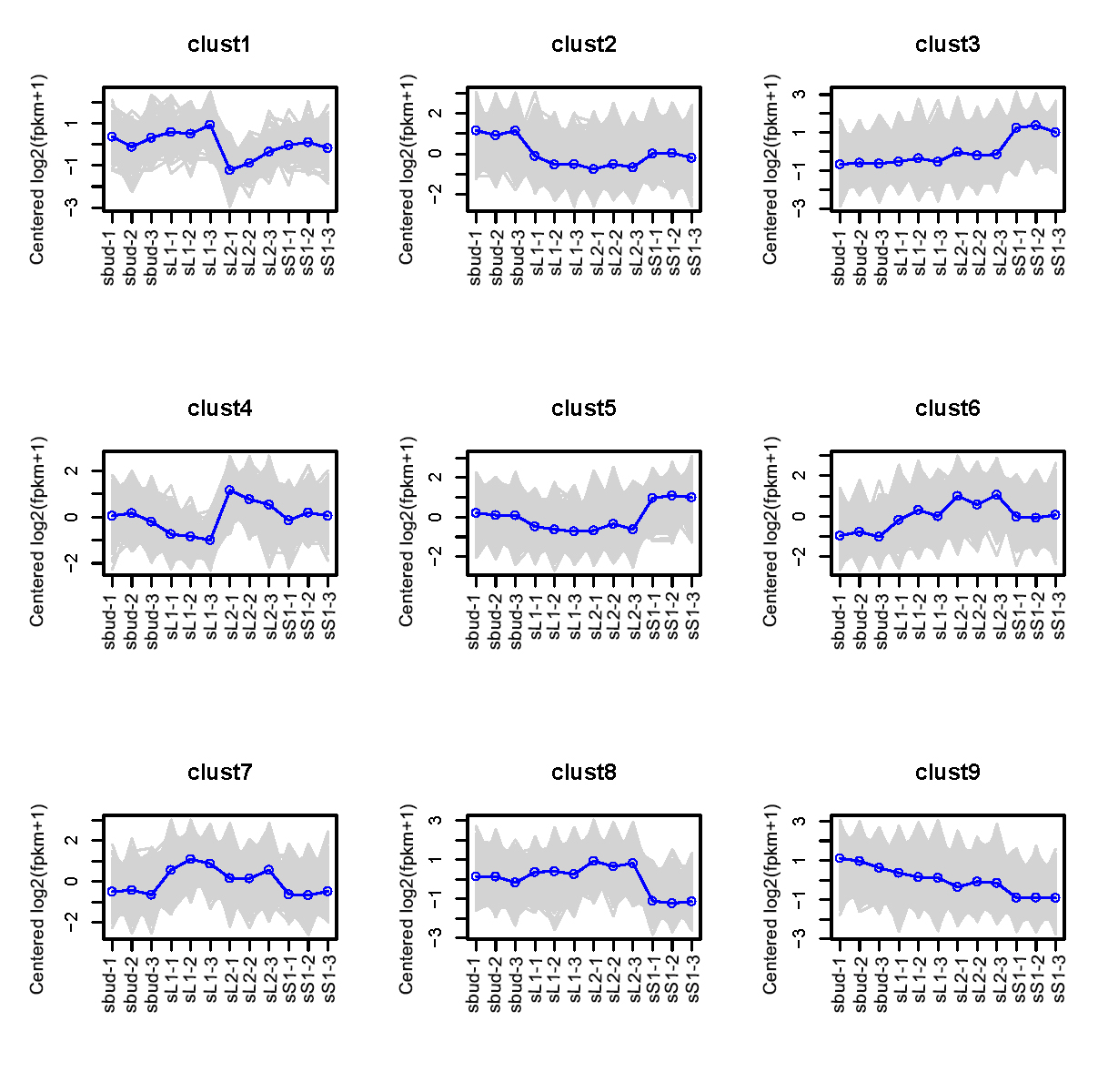

Supplement: Supplementary file 1 [file plants-12-03665-s001.zip › Figure S3.JPEG]

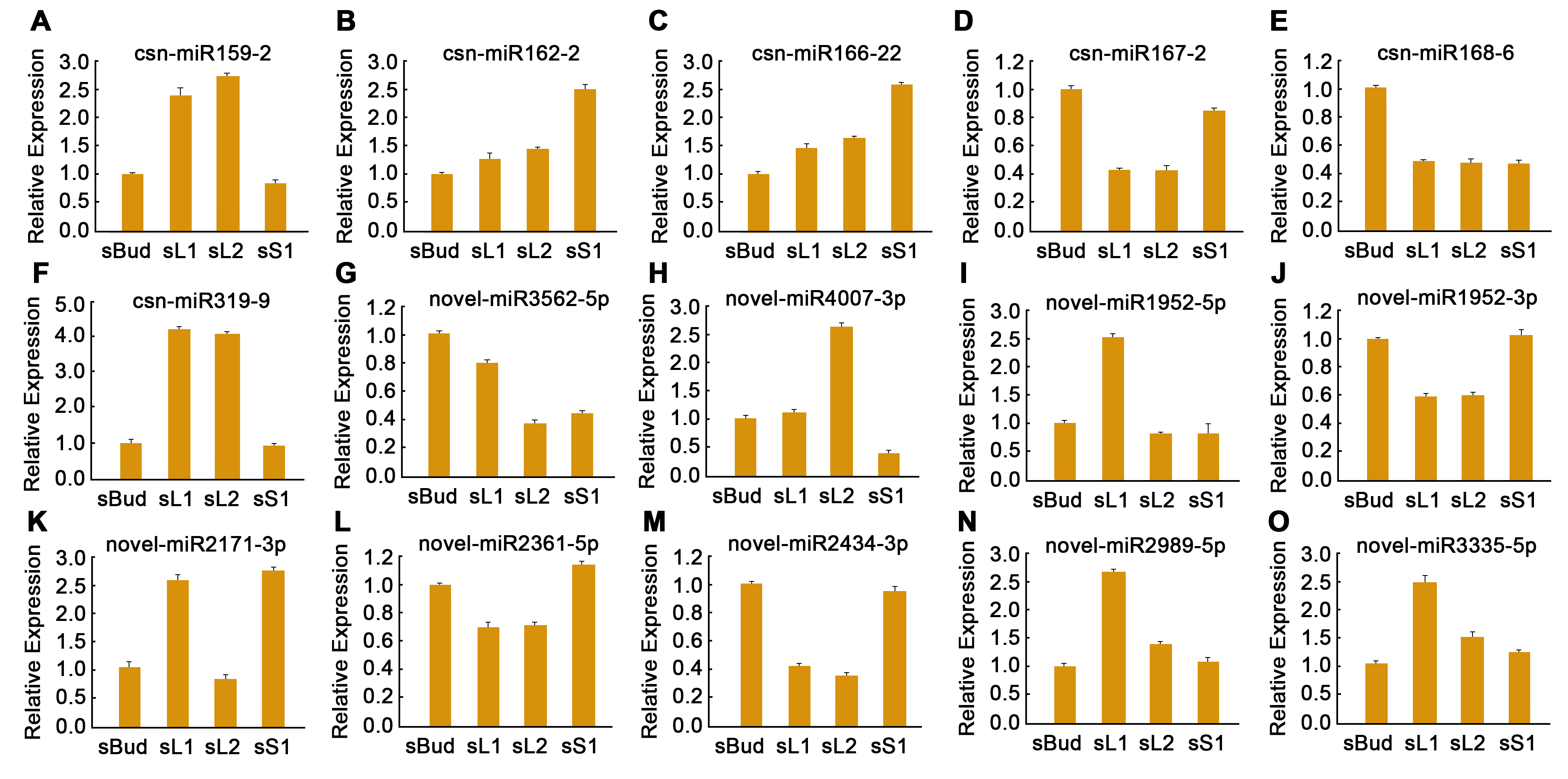

Supplement: Supplementary file 1 [file plants-12-03665-s001.zip › Figure S4.JPEG]

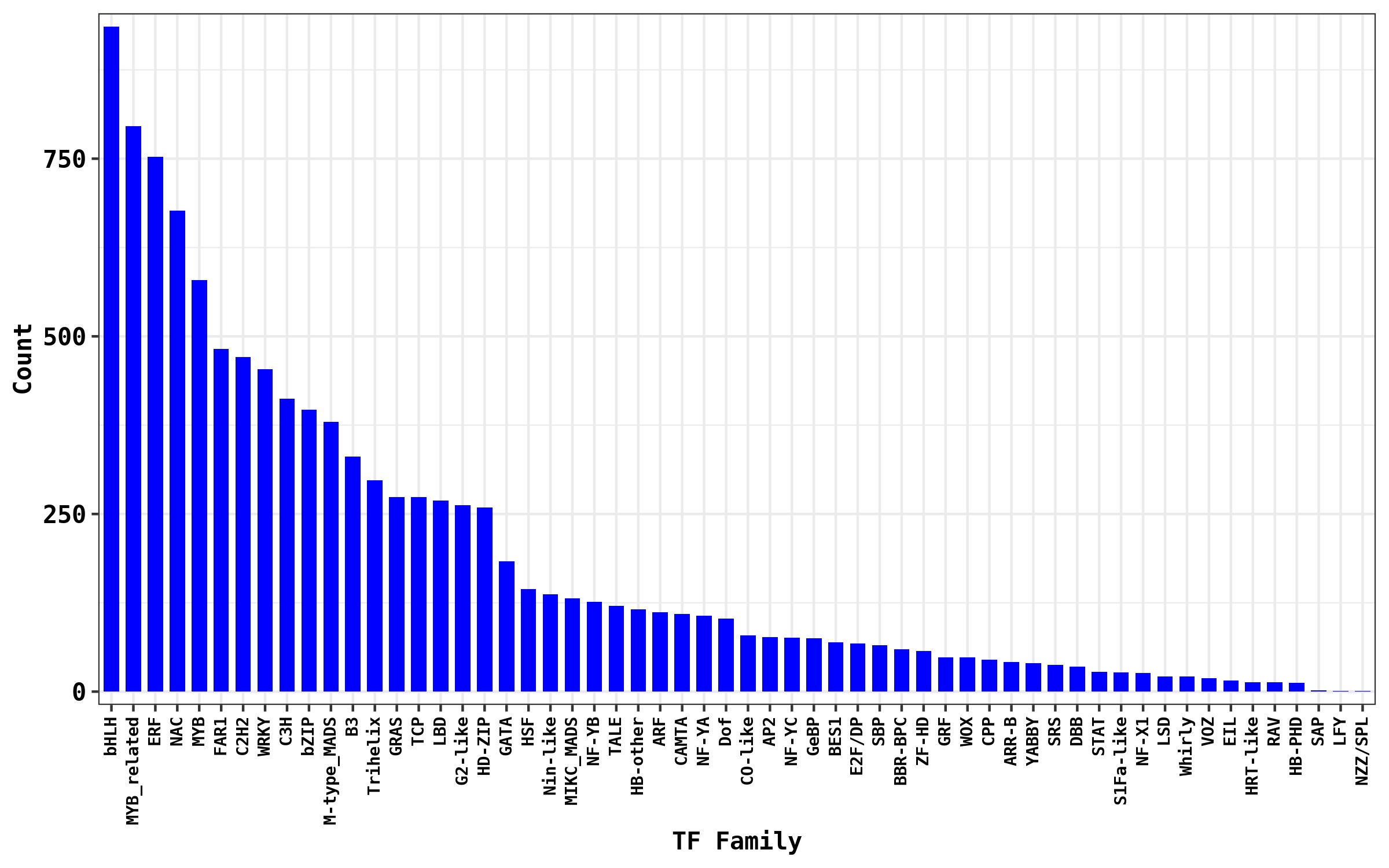

Supplement: Supplementary file 1 [file plants-12-03665-s001.zip › Figure S5.JPEG]
